# Long-term, 13-year survival after immune cell therapy combined with chemotherapy for extensive-stage small-cell lung cancer: a case report

**DOI:** 10.3389/fonc.2024.1389725

**Published:** 2024-06-14

**Authors:** Tong Liu, Heshuang Wang, Qinglong Kong, Haoyu Wang, Haodong Wei, Pengda Sun

**Affiliations:** ^1^ Department of Gastrointestinal Nutrition and Hernia Surgery, the Second Hospital of Jilin University, Changchun, China; ^2^ Department of Central Laboratory, Central Hospital of Dalian University of Technology, Dalian, China; ^3^ Department of Thoracic Surgery, Central Hospital of Dalian University of Technology, Dalian, China

**Keywords:** immune cell therapy, chemotherapy, extensive-stage small-cell lung cancer (ES-SCLC), CD4/CD8 ratio, CIK cell immunotherapy

## Abstract

While the incidence of small-cell lung cancer is low, it has a poor prognosis. Patients with extensive small-cell lung cancer account for about 70% of all cases of small-cell lung cancer, with a median overall survival duration of 8–13 months and a 5-year overall survival rate of only 1%–5%. Herein, we report small-cell lung cancer diagnosed by bronchoscopic biopsy in an adult male patient in 2011. The patient had a clinical stage of cT2N2M1 and stage IV disease (i.e., extensive small-cell lung cancer). Still, he survived for 13 years through a combination of chemotherapy, radiotherapy, and cytokine-induced killer (CIK) immunocell thera. Comprehensive tumor markers, lymphocyte subsets, and lung CT images were obtained through long-term follow-up. After 12 cycles of chemotherapy (CE/IP regimen) and 5940cgy/33f radiotherapy, we found that the patient was in an immunosuppressive state, so the patient was given CIK cell therapy combined with chemotherapy. After 2 years of immunocell-combined chemotherapy, there were no significant changes in the primary lesion or other adverse events. In the 13 years since the patient’s initial diagnosis, we monitored the changes in the patient’s indicators such as CEA, NSE, CD4/CD8 ratio, and CD3+CD4+ lymphocytes, suggesting that these may be the factors worth evaluating regarding the patient’s immune status and the effectiveness of combination therapy. In this case, CIK cell immunotherapy combined with chemotherapy was applied to control tumor progression. With a good prognosis, we concluded that CIK cell immunotherapy combined with chemotherapy can prolong patient survival in cases of extensive small-cell lung cancer, and the advantages of combined therapy are reflected in improving the body’s immune capacity and enhancing the killing effect of immune cells.

## 1 Introduction

Currently, lung cancer has the highest incidence and mortality worldwide among all malignant tumors. Lung cancer is divided into two categories, namely small-cell lung cancer (SCLC) and non-small-cell lung cancer (NSCLC). Small-cell lung cancer accounts for 13–15% of all lung cancers and is an invasive malignant tumor with a poor prognosis associated with undifferentiated and neuroendocrine differentiation of bronchial epithelium. As a highly aggressive neuroendocrine tumor, SCLC is characterized by high growth fraction, short doubling time, and early development of extensive metastasis, resulting in poor prognosis (1). Approximately 30% of all SCLC is classified as localized small-cell lung cancer (LS-SCLC), with a median survival of 16–24 months (2). The remaining 70% are classified as having extensive-stage small-cell lung cancer (ES-SCLC), with a median survival of 8–13 months (3). However, typically, most patients survive less than one year.

Relatively recent research has shown that immunosuppression may be one of the critical factors in the occurrence and development of cancer. Hence, improving the body’s immune capacity through T lymphocyte perfusion may be an essential strategy for anti-tumor activity, and cytokine-induced killer (CIK) cell therapy is a highly suitable choice. As a type of T cell with natural cytotoxic potential similar to NK cells, CIK cells can be induced and differentiated from donor peripheral blood mononuclear cells and are characterized as being non-toxic to normal cells; have wide anti-tumor spectrum (equally sensitive to multi-drug resistant cancer cells) and fast proliferation rate; and show no MHC restriction when T lymphocytes kill. Therefore, it is widely used in cellular adoptive immunotherapy. At present, many studies have shown that CIK cells can achieve good efficacy in the treatment of a variety of malignant tumors (4, 5).

## 2 Case Presentation

The patient was a man in his 40s with a smoking index of 400, who was admitted to the hospital for repeated inflammatory cough. Chest computed tomography (CT) indicated right hilar occupation in the middle lobe of the right lung with solid growth, with suspected lung cancer. PET(PositroEmissionTomography)/CTrevealed right hilar occupation measuring 4.3 cm×3.4 cm×3.8 cm and increased fluorodeoxyglucose extraction of the right hilar and mediastinal lymph nodes. Central lung cancer was considered, with metastasis to the right hilar and mediastinal lymph nodes and double pulmonary nodules. Tracheoscopy biopsy confirmed right lung SCLC, with the following immunohistochemistry findings: CD56(+), Syn(+), P63(-), P40(-), TTF-1(+), CK (+), and Ki-67(+90%). Clinical diagnosis: extensive-stage, right lung SCLC, clinical stage cT2N2M1, stage IV, with metastasis to the hilar and mediastinal lymph node.

**Figure 1:**
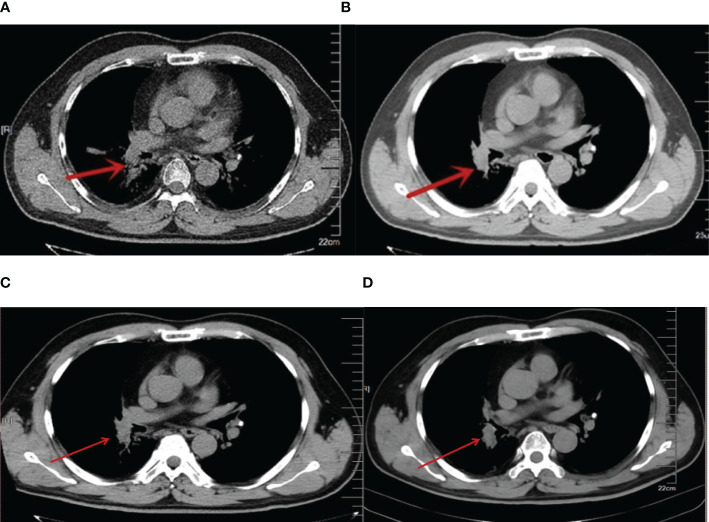
**(A)** 2013 lung CT, red arrow indicating more progressive lesions in the right lung (after chemotherapy). **(B)**. 2013 lung CT, CIK cell therapy combined with chemotherapy after 3 months, the red arrow indicates a reduction in lesions in the right lung. **(C)**. 2016 lung CT. CIK cell therapy combined with chemotherapy. Red arrow indicates lesions in the right lung. **(D)**. 2023 lung CT. Red arrows indicate stable lesions in the right lung.

**Figure 2:**
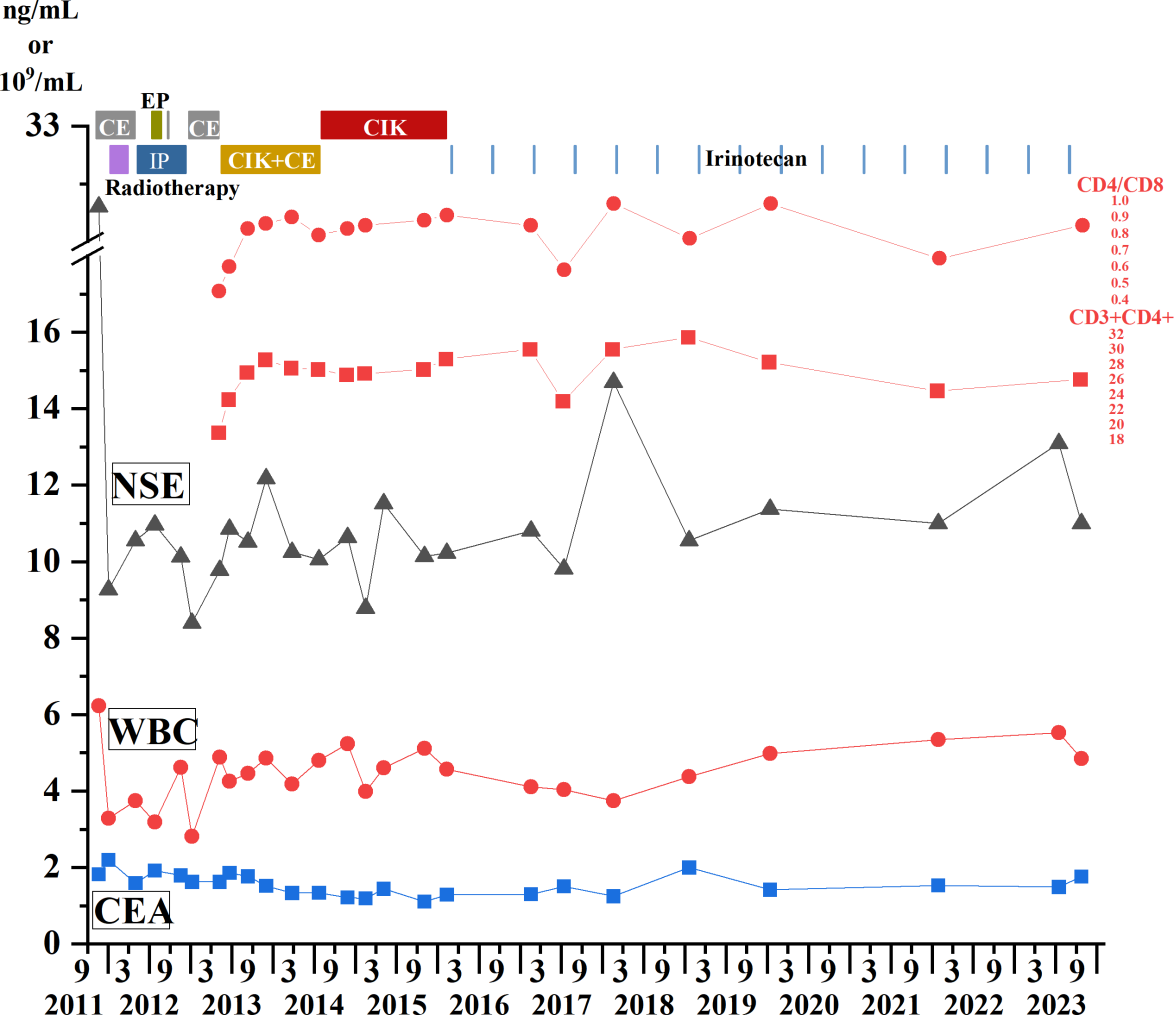
Treatment process from 2011 to 2023. Immune cell therapy, chemotherapy, and radiotherapy are all represented in the upper part of the figure, with different colors and text descriptions representing different treatment modalities displayed in the upper part of the panel. The changes of WBC count and tumor markers CEA and NSE are shown in the line charts. The blue square represents CEA, the red dot represents WBC count, the triangle line chart represents NSE, and the CD3+CD4+ lymphocyte count and CD4/CD8 ratio changes are shown above the NSE line. CEA, Carcinoembryonic antigen; NSE, Neuron-specific enolase; WBC: White blood cell count.

At the beginning of treatment, the patient was given three cycles of standard small-cell chemotherapy and CE regimen every 21 days: carboplatin 500 mg D1 and etoposide 100 mg D1–5. To pursue better therapeutic effect, we simultaneously applied local radiotherapy to complete 5940 cgy/33 f dose of irradiation therapy (a total of two cycles), and then applied the IP protocol in the following 2 years: irinotecan 200 mg D1, D8, cisplatin 30 mg D1 four times; EP regimen 2 times: cisplatin 30 mg D1–3, etoposide 100 mg D1–3; and CE regimen 4 times: carboplatin 500 mg D1, etoposide 100 mg D1–5. The combined application of chemotherapy and radiotherapy had a significant effect in the early stage. With the progression of the disease, the patient’s primary lesion showed signs of growth (**Figure 1A**), and because of the side effects of toxic drugs, the patient showed symptoms such as nausea and vomiting. Lymphocyte subset monitoring showed that the patient was immunosuppressed. Therefore, we consider the application of immune cell therapy to correct the patient’s immunosuppressive state and expect this approach to enhance the effect of chemotherapy. We performed 10 cycles of CIK therapy between 2013 and 2015, and the CIK cell preparation process was as follows: After blood collection, peripheral blood mononuclear cells (PBMC) were isolated from the patient’s whole blood by density gradient centrifugation. Interferon-gamma (IFN-γ,1000 U/mL), anti-CD3 monoclonal antibody (50 ng/mL), Interleukin-2 (IL-2,1000 U/mL) and other cytokines were added *in vitro* for induction. Fresh whole medium was added every 5 days for 14–21 days. The growth of CIK cells was observed under the microscope. Briefly, 2.5×109–5×10^9^ cells were collected and suspended in 100-mL normal saline, and an intravenous infusion of CIK cells was administered three times per cycle. In addition to the CIK cell therapy, we administered CE regimen chemotherapy (carboplatin CBP 300 mg D1, etoposide 100 mg D1–3) for a total of six cycles between 2013 and 2014 in conjunction with immune cell therapy. Four months after CIK immune cell therapy combined with chemotherapy, we reviewed lung CT, and the imaging results showed that the primary lung lesions were significantly reduced (**Figure 1B**). At the end of the treatment cycle of CIK cell therapy combined with chemotherapy, there was no significant progression of the lung lesions (**Figure 1C**). During the treatment process, we monitored the changes of lymphocyte subsets and tumor markers and finally found that the CD4/CD8 ratio and CD3+CD4+ lymphocyte subsets of patients after combined treatment were significantly improved compared with those before treatment. At the most recent monitoring and analysis, the patient’s lymphocyte subsets were still relatively stable. In the subsequent treatment cycle, palliative chemotherapy of irinotecan 100 mg D1 regimen was given according to the patient’s physical state (once every 6 months). In the subsequent monitoring indicators, we found that the patient’s primary lesion was still in a stable state, and the tumor metastasis did not change significantly. No metastatic pleural effusion was observed throughout the course of the disease. More importantly, no signs of distant metastasis were found during the general examination. At the same time, we observed the patient’s WBC and HB indices, both of which were within the acceptable range, and almost no adverse events occurred in this treatment mode (**Figure 2**). In addition to numerical changes in tumor markers and lymphocyte subsets, we were also concerned with changes in the extent of lesions in the lung, as these are valid indicators of therapeutic effect. At the initial stage of disease treatment, given the drug resistance of the tumor, the lesions in the lung tended to increase after stabilizing for a period of time. After a combination treatment of immune cell therapy and chemotherapy, the lesions in the lung were significantly smaller than before. In the latest lung impact data (**Figure 1D**), we could observe that the patient’s lung tumors were stable.

## 3 Discussion

Our patient was first diagnosed with ES-SCLC at the age of 40s. By 2024, he had survived for 13 years with this aggressive disease. The latest intrapulmonary imaging data and tumor evaluation indicators suggest that the disease is stable, with no progression of primary lesions and metastases and no adverse events. To our knowledge, this is the longest survival period in a patient with ES-SCLC treated with CIK immune cell therapy combined with chemotherapy.

When SCLC is unresectable, chemotherapy is preferred as the first-line treatment. However, regardless of the stage of disease at diagnosis, life expectancy and treatment for patients with ES-SCLC have not changed over the past 30 years due to poor prognosis. EP is currently the most commonly used initial chemotherapy regimen for patients with SCLC, and IP is also an option for patients with ES-SCLC (6). Although chemotherapy is effective in the early stages of the disease, most patients with SCLC will eventually relapse and have a short survival. Surgery may be considered for patients diagnosed with stage I or II disease, and is usually combined with chemoradiotherapy. Cisplatin-etoposide is recommended in cases of complete resection with limited or no lymph node involvement (7).

CIK cells can cause tumor cell lysis by releasing granzyme and perforin and can also secrete a variety of cytokines such as IFN-γ, TNF-α, and IL-2, which can not only directly inhibit tumor cells but also indirectly kill tumor cells by regulating immune system reactions. In addition, the targeted migration of CIK cells to tumors, tumor-associated lymph nodes, and spleen tissues also enhanced the cytotoxic potential of CIK cells (8). To enhance the cytotoxicity of CIK cells, in addition to combination chemotherapy, CIK cells are often used clinically with other cytokines (e.g., IL-6, IL-7, IL-15); dendritic cells; immune checkpoint inhibitors; specific antibodies; and Car(Chimeric antigen receptor) T-cell therapy (9).

Currently, immunotherapy is a third-line strategy for single-drug treatment of SCLC, and many trials are exploring the possibility of immunotherapy combined with chemotherapy as the first-line treatment of SCLC (10). The results of some recently published clinical trials show that the combination of immune checkpoint inhibitors and chemotherapy has a good effect in the treatment of ES-SCLC, providing more reliable evidence. Overall survival (OS) and progression-free survival (PFS) were significantly better with combination therapy than with chemotherapy alone in ES-SCLC (11, 12). However, in SCLC, immunotherapy is less effective than in other solid tumors, such as NSCLC, because this highly malignant tumor has multiple immune escape mechanisms, and only a small percentage of patients experience lasting benefit from treatment.

CIK immunotherapy, in combination with traditional therapies, has played an essential role in controlling the progression of the disease. Clinical study have shown that CIK immune cell therapy can be used as a maintenance therapy for SCLC, which can help in prolonging survival (13). In addition, CIK immune cells have been reported to have a synergistic effect when combined with conventional therapies such as chemotherapy and immune checkpoint inhibitors (ICIs), enhancing anti-tumor effects with few serious adverse events (14). A 2016 clinical study of ES-SCLC showed that CIK cell therapy combined with chemotherapy had a significant advantage over chemotherapy alone in terms of overall response rate and PFS (15). Some studies have shown that chemotherapy can prevent tumor-induced immunosuppression by activating immune effector cells such as natural killer cells (16). In addition, chemotherapy drugs can cause antigen release of tumor cells through cytotoxic effects. By exposing more antigen surface sites, the sensitivity of immune cells can be up-regulated, the effect of immune cell therapy can be enhanced, and cytotoxic T cells can better enter the tumor microenvironment to kill tumor cells (17, 18). Moreover, CIK cells can reduce bone marrow suppression caused by chemotherapy, enhance immune status, eliminate chemotherapy resistance, and even directly kill tumor stem cells (19, 20). The application of chemotherapy drugs can improve the anti-tumor effect of immune cells, promote the differentiation of CD8+ T memory cells into CD8+ T effector cells, and enhance the enrichment of immune cells into the tumor microenvironment (21). Follow-up CIK immune cell therapy can directly kill tumor cells and eliminate tumor drug resistance, thereby improving prognosis.

For medium- and advanced-stage SCLC patients, the long-term effect of treatment is poor because of the drug resistance and immunosuppression of the disease. The patients in this study received relatively systematic chemotherapy and radiotherapy, and the changes in lymphocyte subsets and tumor scope were monitored in a timely manner after the subsequent progression of lung lesions. The decrease of CD3+CD4+ lymphocyte count and CD4/CD8 ratio, as well as the increase of lung lesions, indicated tumor progression and immunosuppression. We effectively relieved the immunosuppressive state of the patient by applying CIK cell therapy in time, and after combining it with chemotherapy, the tumor progression was very effectively controlled. It was evident that after the application of immune cell therapy combined with chemotherapy, the results of lymphocyte subsets showed that the immunosuppressive state of the patient was effectively improved, and the scope of the lesion seen in the lung CT image was significantly less than before. More importantly, in the subsequent long-term follow-up process, tumor markers, CD3+CD4+ lymphocytes and CD4/CD8 ratios, and the extent of lung lesions were all in a very stable state. The CD4/CD8 ratio is one of the important indicators reflecting the immune status of the body, and the decrease of CD4/CD8 ratio is closely related to immunosuppression and poor prognosis (22). At present, most studies focus on the interaction between CD8+ T lymphocytes and the tumor microenvironment, but neglect the vital role of CD3+CD4+ Th cells in coordinating innate and adaptive immunity (23). It has been reported that specific CD4+T lymphocyte populations also mediate tumor killing and regression (24) and can also be used as one of the indicators reflecting the immune status of tumor patients. Monitoring the changing trend of CD4/CD8 ratio and CD3+CD4+ lymphocytes during follow-up can be used as an effective indicator to reflect the immune status of cancer patients during treatment.

## 4 Conclusions

Herein, we report a 13-year, long-term survival event in a patient with ES-SCLC who was treated with CIK cell therapy combined with chemotherapy, resulting in the lesion remaining stable over a long period. We believe that early assessment of the patients’ immune status is critical, and the combination of immune cell therapy and chemotherapy should be carried out as soon as possible to relieve the immunosuppressive state and reverse tumor drug resistance. Immune cell therapy combined with chemotherapy may be an effective treatment for patients with ES-SCLC. The application of immune cell therapy at the right time can alleviate the lymphocyte failure caused by long-term chemotherapy and increase the lymphocyte-killing effect. CD4/CD8 ratio, CD3+CD4+ lymphocyte count, tumor markers, and imaging changes of lesions were used to estimate treatment effectiveness and long-term prognosis of cancer patients.

This case report suggests the effectiveness of immunotherapy in the treatment of extensive-stage SCLC and highlights the potential of CIK cell therapy in combination with chemotherapy as an emerging therapy in the treatment of extensive-stage SCLC.

## Data availability statement

The original contributions presented in the study are included in the article/supplementary material, further inquiries can be directed to the corresponding author/s.

## Ethics statement

The studies involving humans were approved by ethics committee of Central Hospital of Dalian University of Technology. The studies were conducted in accordance with the local legislation and institutional requirements. Written informed consent for participation in this study was provided by the participants’ legal guardians/next of kin. Written informed consent was obtained from the individual(s) for the publication of any potentially identifiable images or data included in this article.

## Author contributions

TL: Writing – original draft, Writing – review & editing. HeW: Conceptualization, Writing – review & editing. QK: Data curation, Writing – review & editing. HWa: Conceptualization, Data curation, Writing – review & editing. HaW: Writing – review & editing. PS: Conceptualization, Data curation, Funding acquisition, Supervision, Writing – original draft, Writing – review & editing.
